# An Immersive Motor Protocol for Frailty Rehabilitation

**DOI:** 10.3389/fneur.2019.01078

**Published:** 2019-10-15

**Authors:** Elisa Pedroli, Pietro Cipresso, Luca Greci, Sara Arlati, Lorenzo Boilini, Laura Stefanelli, Monica Rossi, Karine Goulene, Marco Sacco, Marco Stramba-Badiale, Andrea Gaggioli, Giuseppe Riva

**Affiliations:** ^1^Applied Technology for Neuro-Psychology Lab, Istituto Auxologico Italiano - Istituto di Ricovero e Cura a Carattere Scientifico, Milan, Italy; ^2^Department of Psychology, Università Cattolica del Sacro Cuore, Milan, Italy; ^3^Institute of Intelligent Industrial Technologies and Systems for Advanced Manufacturing, National Research Council, Milan, Italy; ^4^Department of Electronics, Information and Bioengineering, Politecnico di Milano, Milan, Italy; ^5^Department of Geriatrics and Cardiovascular Medicine, Istituto Auxologico Italiano - Istituto di Ricovero e Cura a Carattere Scientifico, Milan, Italy

**Keywords:** motor rehabilitation, virtual reality, CAVE, frailty, elderly, stationary bike, balance, risk of falls

## Abstract

Frailty is a pre-clinical condition that worsens physical health and quality of life. One of the most frequent symptoms of frailty is an increased risk of falling. In order to reduce this risk, we propose an innovative virtual reality motor rehabilitation program based on an immersive tool. All exercises will take place in the CAVE, a four-screen room with a stationary bike. The protocol will include two types of exercises for the improvement of balance: “Positive Bike” and “Avoid the Rocks.” We will choose evaluation scales related to the functional aspects and subjective perception of balance. Our aim is to prove that our innovative motor rehabilitation protocol is as effective as or more effective than classical rehabilitation.

## Introduction

The constant increase of the elderly population compared to other age groups is now an evident phenomenon (1) which has led to increased efforts to propose solutions to the problems arising from the physiological condition of the elderly. Aging causes changes in both cognitive and motor functioning, which, depending on the degree of decline, can impact on different aspects of life with repercussions at various levels. In particular, it is possible to outline a condition of particular vulnerability in a part of this population, in patients defined as “frail,” which represent 6.9% of adults over 65 years old (2). In this pre-clinical condition, there is a pattern of decline in the functioning of different aspects such as gait, mobility, balance, and cognitive functioning (3). These aspects associated with increasing age place these patients in a particular condition of vulnerability that is directly associated with a high risk of adverse health outcomes, mortality, disability, and more commonly a higher risk of falls (2, 4–6). The diagnostic criteria for this condition are: unintentional weight loss (10 lbs in the past year), self-reported exhaustion, weakness (grip strength), slow walking speed and low physical activity. Three or more of these criteria are needed for diagnosis according to the definition of Fried and colleagues (2).

Among the consequences of frailty mentioned above, the risk of falling is one of the most frequent and critical health problems occurring in the elderly and in particular in the frail population. It is estimated that one out of three elderly people falls at least once a year (7). This event has important consequences both for the autonomy of the individual and for problems in the psychosocial area, with further repercussions for cognitive functioning and quality of life (8, 9). The risk of falling in old age is a phenomenon that can be explained on the basis of the interaction between cognitive and motor factors. In fact, correct locomotion presupposes the possibility of simultaneously managing gait performance and one or more cognitive tasks (10). The presence of cognitive activity during the execution of motor tasks often occurs in daily activities (11, 12). The possibility of performing both tasks concurrently could be compromised in the elderly, who often show a decrease in attention skills and executive functions, thus leading to an increased risk of falls.

This phenomenon is interpreted according to the cognitive-motor interference (CMI) theory (13, 14), which states that the simultaneous execution of a motor task and a cognitive task represents a kind of dual-task (DT) interference that requires great cognitive resources and in particular attentive abilities and executive functioning (15). Depending on the complexity of the cognitive task, its simultaneous execution with motor performance may compromise the execution of motor performance, of cognitive performance or both (1, 15). However, on the basis of the information we have, DT tasks have been tested and proved to be an excellent tool for the simultaneous treatment of motor and cognitive abilities, and consequently for the recovery of abilities (such as gait) and the reduction of the risk of falls.

On the other side, work on balance separately is important in order to provide more focused exercises. A recent review (16) underlay the importance of balance in reducing the risk of falls in the elderly. In particular, old subjects with deteriorated balance fall more frequently than seniors with unimpaired postural control, which emphasizes the need for balance and postural training in this specific population (17). Almost all the studies that have investigated the prevention or the treatment of the risk of falling in the elderly conclude that different kinds of physical activity are effective for balance control and fall prevention (18). Osoba et al. (19) strongly recommend treatments to improve balance and gait in the elderly, in particular with virtual reality.

Considering frailty as a dynamic and reversible process with transition between states over time (20), many studies have focused on the possibility of reducing frailty and the risk of falls with specific interventions and activities that can prevent this condition (21). The motor rehabilitation approach based on physical exercise, both aerobic and to increase strength (22), has proved useful in reducing the risk of falls (23–26) and for the general improvement of cognitive functioning (27). Physical exercise, such as balance, strength, flexibility, and coordination training, is associated with a reduction in the risk of falls not only in healthy elderly people but also in individuals with cognitive impairment (28) and specifically in frail older people (23, 24). A one-size-fits-all program is not suitable, as the intensity of the exercise must be proportional to the patient's capabilities (29). But with regard to the kind of treatment, a recent systematic review showed that exercises to increase strength and postural balance are those most associated with the prevention of falls (25). Moreover, specific treatments for the training and recovery of balance mechanisms, such as treadmill-based systems, therapist-applied perturbations and perturbation-based balance training, would be more effective than general exercises (30, 31).

Several studies suggest the effectiveness of the integration of motor and cognitive training to decrease the risk of falls, and the DT approach seems to be one of the more efficient for the improvement of motor and cognitive abilities (29, 30). The contribution of higher-order cognitive systems such as executive functions makes this approach an effective training for the treatment of fall risk (10).

Virtual reality (VR) has improved the development and implementation of interactive cognitive-motor training programs. Ecological and realistic environments can be created by means of VR, which depict real/daily life situations with beneficial effects on patients' acceptance and adherence (32). Balance and functional mobility are the main domains tackled by VR with promising outcomes, suggesting this tool as an appropriate rehabilitative approach (33).

According to positive technology theory (34), interaction with technology leads to positive emotions and self-growth. The quality of psychological intervention can benefit from what is called “transformation of flow.” According to Riva and colleagues, the user is able to exploit the optimal experience with VR and increase his/her involvement to obtain better performances (35, 36). Thanks to VR is possible to create a task both involved and challenging in order to engage patients, leading to promising results in cognitive and physical rehabilitation (37, 38).

VR cycling training for motor rehabilitation has been used in old adults and stroke patients (39–43); however, no one has implemented DT protocol with physical and executive functions. We will describe the rationale, design and usability of a fully-immersive VR DT biking navigation called the “Positive Bike.” To our knowledge, the majority of the research on balance training involves standing posture; fewer studies have focused on sitting posture rehabilitation (42). Stationary cycle exercises have a positive effect on weight shifts and gait, as well as the functioning of lower body limbs and a reduction of fall risk (44–46). Cycling also contributes to maintenance of specific balance coordination patterns and could help to preserve balance control and speed of voluntary stepping in the elderly (47). Walking is very close to cycling; indeed, they are both cyclical and activate agonist-antagonist muscles (48–50). Additionally, stationary cycling provides a controllable workload and safer equipment compared to the treadmill, leading to lower risk of injury in frail users (51).

Accordingly, in this paper an innovative VR-based protocol is proposed. The aim of the training will be to increase balance in frail people so as to reduce the risk of falls. This protocol will be developed within a national financed project with the purpose of creating both high- and low-end tools for motor (52, 53) and cognitive rehabilitation (54, 55). In this paper we will focus on the high-end motor part.

## Motor Rehabilitation Training

### Inclusion/Exclusion Criteria

The eligibility criteria will require the participants to be 65 years of age and older, to match at least three of the five frailty criteria of Fried and colleagues (2) and to have an MMSE (56) score between 30 and 27. Fried's criteria include unintentional weight loss, self-reported exhaustion, weakness, slow walking speed and low physical activity. Exclusion criteria include significant vision impairments, presence of depression or anxiety without medications and hemianopsia or hemiplegia. The presence or absence of these criteria will be assessed during the initial clinical assessment performed by a physician. If patients report some depression or anxiety symptoms or the clinician suspect one of this problems he will be given specific tests, like Beck Depression Inventory [BDI-II (57)] or the State-Trait Anxiety Inventory [STAI Y1-Y2 (58)]. The final sample will be composed of 64 patients; in order to achieve this goal, at least 80 subjects will be assessed. To evaluate the size of the involved samples, we will use a Sample Size Calculation (Power Analysis) using the software GPower^*^3. The recent randomized trial assessing reduction in frailty (59) found that changes in frailty and mobility are similar in magnitude and represent medium effect sizes. Using their data, we estimated a minimum of 64 subjects to be included in the physical rehabilitation experiment in order to achieve a minimum power of 90%, considering a medium effect size of 0.4, a 15% dropout/non-compliance rate and a significance level of 0.05. Possible side effects connected with VR systems, such as nausea or dizziness, are referred to as cybersickness.

### Outcome Measures and Data Analysis

We will choose evaluation scales related to the functional aspects (points 1, 2, and 3) and the subjective perception of balance (point 4). We will also take objective data using the Neurocom Balance Master (point 5). A general muscle strength assessment (point 6) will take using a hand grip dynamometer, used also for testing the frailty of the patients. A trained physiotherapist performed the assessment in order to avoid low reliability of the data. All the information are included in the Table 1.

1. The *Tinetti Balance Scale* (60) is considered a gold standard for the validation of balance tests. It is a simple clinical that consists of 14 items with a score out of 24. The higher the score, the better the performance.
2. The *Equiscale* (61) takes into account the three subdomains of still standing, resistance to external perturbations and resistance to self-induced perturbation. Real-life performances, such as leaning forward and sitting up, are represented.
3. The *Timed Up and Go Test* (TUG) (62) takes into consideration the time that the subject takes to get up from a standard chair, walk three meters, turn around and go back to sitting down. The time was measured from the moment the clinician says “go” to the moment the participant sits back in the chair.
4. The *Dizziness Handicap Inventory* (DHI) assesses the self-perceived handicapping effects of balance system disease (63). The DHI consists of 25 items derived from three content domains believed to encompass the functional, emotional and physical impacts of balance system disease. The subject can answer in three different ways (Yes, No, Sometimes) for each question. The “yes” response is scored 4 points, the “sometimes” response is scored 2 points, and the “no” response is scored 0 points. High score relates to high impact of the symptoms on the patient's daily living.
5. The Neurocom Balance Master® (64) uses a fixed force plate to measure the vertical forces exerted through the patient's feet to measure center of gravity position and postural control. With this instrument we can take objective measures of: (1) the modified Clinical Test for the Sensory Interaction on Balance (CTSIB), which estimates balance by measuring the speed of oscillation of the Center of Pressure (CP) with open then closed eyes and firm then mossy ground; (2) limits of stability: the possibilities of moving the CP toward a predetermined target without moving the feet; and (3) rhythmic weight shifting in the frontal and sagittal plane, without moving feet. These data have clinical significance because they give us numerical data about the ability of the subject to maintain balance in standing and static position tests.
6. The *strength* outcome (65) was measured as the best performance of three readings using a handheld dynamometer (Jamar, Sammons Preston, Bolingbrook IL). We tested both hands.

**Table 1:** Outcome measurements.

**Test**	**Outcome informations**	**Administration**	**Primary outcome**
Tinetti Balance Scale	Balance	Assessment scale compiled by the physiotherapist	
Equiscale	Standing and resistance to external and self-induced perturbation	Assessment scale compiled by the physiotherapist	
TUG	Mobility	Assessment recorded by the physiotherapist	
DHI	Self-perceived balance	Questionnaire compiled by subject	
Neurocom Balance Master®	Objective measures of balance	Information recorded by a software	*
Handheld dynamometer	Strength	Assessment recorded by the physiotherapist	

Our hypothesis is that our VR rehabilitation program is more effective than classic treatment in improving objective and subjective outcome measures. In order to confirm our hypothesis, we will perform Mixed Model ANOVA to compare the difference between the groups (VR VS NN-VR) and also between the time (T0, T1, T2) for each outcome measure collected. Also, the Bayes Factor will be used to determine if our program is more effective than classic treatment.

### Protocol

**Figure 1:**
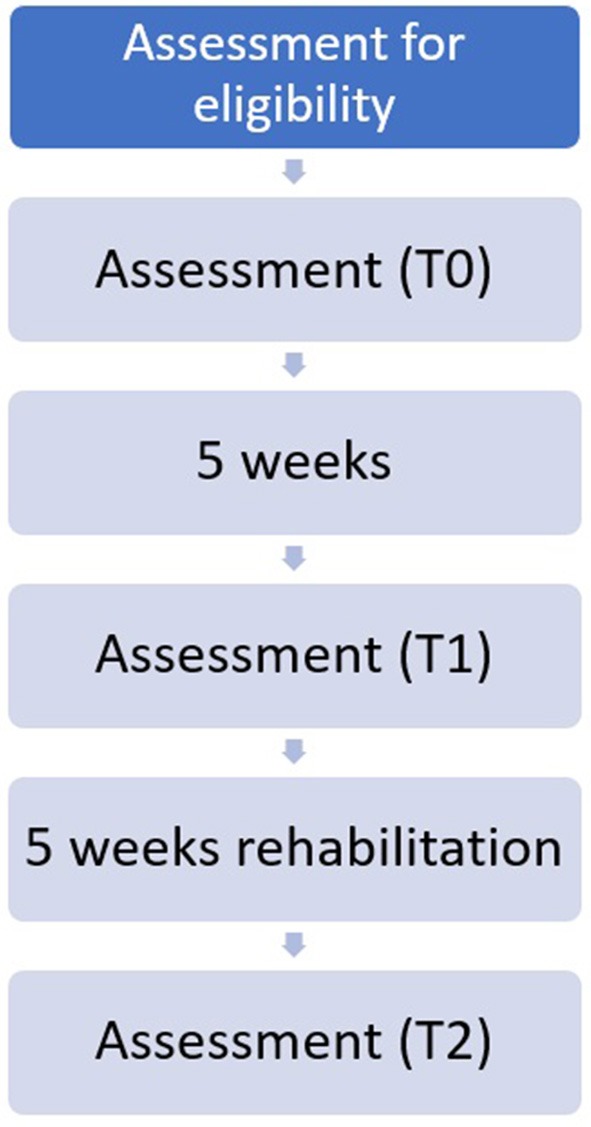
Work flow.

During the first medical examination, the inclusion and exclusion criteria were assessed by a physician. If the subject was considered suitable for the clinical protocol, outcome measures will be collected (T0) by a trained physiotherapist. Patients will be then randomly assigned to a control or experimental group using a randomization sequence obtained from the site randomizer.org. The first group will undergo classical physiotherapy, while the other one started a VR protocol. After 5 weeks without physical treatments, patients will return to the hospital to undergo a second evaluation (T1). Then, 10 biweekly rehabilitation sessions will start, and at the end a new assessment will be done (T2). The workflow is presented in Figure 1. Each session will last approximately 45 min and included both cycloergometer and dynamic exercises. To consider the treatment valid, patients will have to participate in at least 8 of 10 rehabilitation sessions and all the assessments; patients who will execute fewer than eight sessions will be considered drop-out (Figure 1). All participants will sign the written informed consent, which was approved by the Ethical Committee of IRCCS Istituto Auxologico Italiano. The study was conducted in compliance with the Helsinki Declaration of 1975, as revised in 2008.

## VR Settings

The training will take place in a Cave Automatic Virtual Environment (CAVE). The CAVE system consists of a room-sized cube in which a combination of four stereoscopic projectors (Full HD 3D UXGA DLP) is used to obtain a 3D visualization of the virtual environment (VE) scene onto three walls, plus the floor. The projected right-eye and left-eye images are combined together by active goggles, making the perception of depth possible. In addition to the visualization devices, CAVE is equipped with an optical tracking system (VICON). Such a system allows the tracking of passive reflective markers and enables the correction of the spatial distortion of the simulated environment, which is eventually displayed in the CAVE with a 1:1 scale ratio. In our study, both CAVE goggles and an Xbox joystick are equipped with an asymmetrical set of markers allowing for the retrieval of their position and heading in the space. These pieces of data are used, respectively, to adjust the user's point of view and to enable the use of the Xbox joystick as a pointer for the interaction with 2D interactable elements (i.e., buttons) in the CAVE.

All the CAVE functionalities are handled by a cluster system composed of two HPZ620 Graphics Workstations, mounting Nvidia Quadro K6000 GPU with dedicated Quadro Sync cards.

**Figure 2:**
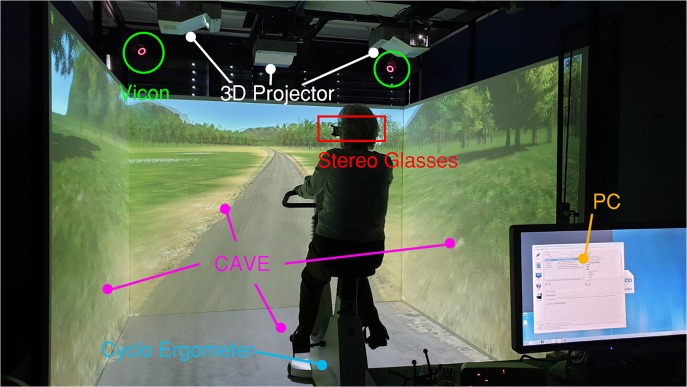
The VR system, CAVE.

Both VEs described in the following paragraphs were developed using Unity 3D and MiddleVR Unity plug-in. Thanks to this plug-in, the application deployed from Unity can communicate with all the CAVE system modules: the scene can be projected onto the CAVE walls, and the motion data retrieved from the VICON system can be exploited as inputs. The parts of the system are highlighted in Figure 2.

### Stationary Bike

The Positive Bike application requires a stationary bike (Cosmed EuroBike 320) placed inside the CAVE. Bike velocity and workload can be, respectively, read and set–via a serial cable–thanks to an *ad-hoc* developed protocol exploiting the bike manufacturer's Software Developing Kit (SDK). A pushing button is anchored on the cycloergometer handlebars for the detection of user interaction, and an Arduino2 board is used to connect the button to the computer.

Besides the GUI (graphical user interface), which is dedicated to the operator for the exercise parameters setting, the application is composed of two parts. The first one represents a trail in a park that flows according to the pedals' velocity (measured by the cycloergometer in revolutions per minute, RPM). The user bikes along the predefined path, which is created thanks to the placement of subsequent nodes on the route; the interpolation of such nodes is performed in real time using quaternion spherical linear interpolation (Slerp).

Since the user cannot deviate from the predefined route, the park is designed to discourage any desire to turn: there are no road forks, and around the unpaved path there is just grass. To avoid boredom, some elements of the landscape change throughout the exercise, e.g., different species of plants and trees, lakes, buildings, etc. appear in the background. The path has some bends to increase the realism of the scene, but they are all very slight to avoid the occurrence of cybersickness related to the expectations of a lateral acceleration. Some tests made before this study ensured that no cybersickness arose in healthy subjects because of the bends.

During the exercise, participants are asked to keep their cycling velocity between 55 and 65 RPM. The bike workload is set by the therapist at the beginning of the exercise according to the subject's physical status. If the biking velocity is too low or too high, audio feedback is provided to the user: an acute sound is reproduced to signal to the user that he/she has to slow down; a grave sound is used to ask to speed up. The choice of signaling errors related to the physical part of the DT training was made to avoid distracting the elderly from the cognitive task by introducing an additional visual feedback.

**Figure 3:**
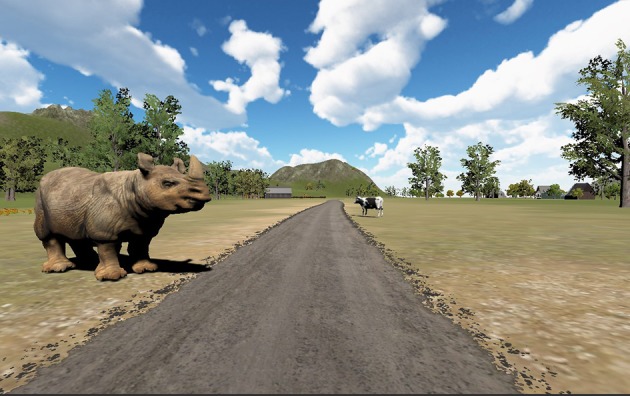
A frame of the “Positive Bike” environment.

The cognitive task of the exercise foresees the recognition of targets (Figure 3) appearing randomly on either the left or right side of the biking path. Targets are animals whose names start with a predefined letter that is communicated to the patients prior to the exercise beginning; other animals are considered distractors. The time elapsing between two subsequent targets is decided by the therapist who sets the exercise parameters. All the targets appear when the user is at a distance of 20 meters, so that he/she can clearly discriminate the targets' features. Target selection occurs by pressing the button placed on the cycloergometer handlebar while the target is still in the subject's visual field (i.e., it is displayed on the right or left wall of the CAVE).

Each time the user presses the button, he/she receives visual feedback regarding the correctness of the choice. No feedback is given if the user does not press the button, either if the choice is correct (the displayed object is a distractor) or if the target has been missed. All data related to the cognitive exercise execution, as well as all the parameters set by the therapist, are stored in a user-dedicated folder in XML format.

The second part of the training occurs at the end of the biking: the screen displays a written question asking the subject how many targets he/she remembers having picked. The therapist types the answer on the CAVE computer's keyboard and saves this piece of information together with the exercise data saved at the end of the previously described scenario.

### Avoid the Rocks

**Figure 4:**
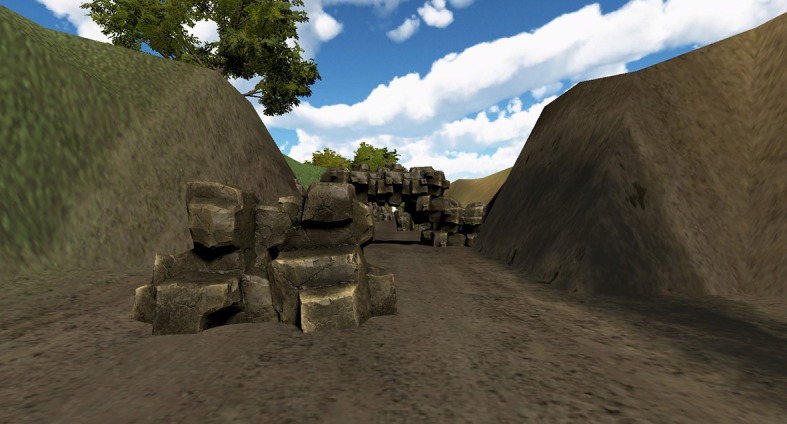
A frame of the “Avoid the Rocks” environment.

The aim of this VE is training balance in frail people by using a virtual environment running in a CAVE. The VE simulates a walk on a straight road. Along the road, the user encounters obstacles (i.e., different-shaped rocks) and has to avoid them. Obstacles are positioned on the road so that the user is stimulated to perform lateral movement (left and right) or to bend down to avoid hitting the rocks (Figure 4).

The user does not need to walk to proceed forward since his/her walking is simulated by displacing the user's point of view in the forward direction. The speed of the displacement can be increased or decreased by the operator pushing the “+” or “–” buttons on the keyboard.

To allow for the detection of collisions between the user and the rocks along the path, a virtual model of the player (i.e., a capsule-shaped object) is used. Such a model follows the user's head displacement in the CAVE, which is measured in real time thanks to the tracking of the 3D goggles. When the virtual model of the player is detected as colliding with an obstacle, it triggers the reproduction of a sound signaling the error. Similarly to the previous scenario, data regarding users' performance are stored in an XML file in a user-dedicated folder.

## Classical Rehabilitation

The training will take place in the rehabilitation gym of our hospital under the supervision of a physical therapist. In order to replicate the protocol proposed in the virtual rehabilitation, we developed two groups of tasks, one with the stationary bike and the other with classic balance tasks, as described below. Each part require 15 min, all the sessions are about 30–40 min according to the needed of the patients.

### Stationary Bike

We will use a stationary bike (the same model used in the VR protocol) placed in the gym. The therapist set the workload, increasing it session by session according with the training level gained by the subject. During the exercise, participants are asked to keep their cycling velocity between 55 and 65 RPM. No dual task was required.

### Balance Training

We will use a training protocol specific to balance in every subject. In literature, no specific tasks for increase balance in frail elderly people are reproted. We will use some devices such as a balance pad, proprioceptive footboard, rocking footboard, etc. for exercise mono- and bi-podalic station. We will train the subjects with and without visual deprivation. The workload is regulated according to the physical status and performance ability of the subject. Example the therapist ask to patients to mantein balace standing on one foot with the arms cross on the chest.

## Discussion

The aim of this VR rehabilitation protocol will be to improve balance and reduce the risk of falls in frail elderly people. In order to assess these hypotheses, we will develop an innovative tool using an immersive VR system, the CAVE. We will compare this innovative protocol with a selection of classical physiotherapy exercises with the same purposes.

### Expected Results and Limitations

According to our hypotheses, we would like to prove that our innovative motor rehabilitation protocol is as effective as or more effective than classical rehabilitation. We will include both subjective and objective measures in order to better understand the degree of improvement subjects will obtain. Positive Bike aims to improve the dual-task abilities of frail elderly people using an innovative, engaging and challenging training. We hope that both the subjective and objective measures will increase after our training.

### Future Steps

Several studies (66–68) have showed that continuing rehabilitation activities at home contributes to the maintenance of benefits obtained. Accordingly, we are developing a low-end VR tool to promote physical rehabilitation at home. This new system will be tablet-based and will exploit the potential of 360° videos (68–70). To our knowledge, there are no studies that have tested this technology for motor rehabilitation. 360° videos are usually enjoyed by using head-mounted displays. We decided to use tablets instead of head-mounted displays to reduce the risk of injuries. Performance of balance exercises excluding patients from the “real environment” could be risky, and tablets are a safer tool for the use of 360° videos. We will try to replicate the protocol used for rehabilitation in the high-end VR setting by adapting it to low-end technology. We will also provide the patients with a portable cycloergometer in order to perform the dual-task exercise.

## Author Contributions

EP, PC, LB, MR, LS, and KG conceived and designed the protocol. LG and SA participated in the design, developed the environments, and integrated the cycloergometer functionalities. EP and LG wrote the first draft. MR provide the required revisions. MS, MS-B, GR, and AG are supervisors. All the authors revised the final version of the manuscript.

### Conflict of Interest

The authors declare that the research was conducted in the absence of any commercial or financial relationships that could be construed as a potential conflict of interest.
